# Uncertainty and novel clinical trial designs in oncology – when should equipoise matter?

**DOI:** 10.1177/17407745261469887

**Published:** 2026-09-11

**Authors:** Sarah Heynemann, Wendy Lipworth, Sue-Anne McLachlan, Jennifer Philip, Tom John, Ian Kerridge

**Affiliations:** 1 Sydney Health Ethics, Faculty of Medicine and Health, The University of Sydney, NSW, Australia; 2 Department of Oncology, Northern Health, Melbourne, VIC, Australia; 3 Department of Philosophy, Macquarie University, Sydney, NSW, Australia; 4 Department of Medical Oncology, St Vincent’s Hospital, Melbourne, VIC, Australia; 5 Department of Medicine, Faculty of Medicine, Dentistry and Health Sciences, The University of Melbourne, VIC, Australia; 6 Department of Palliative Care, St Vincent’s Hospital, Melbourne, VIC, Australia; 7 Department of Palliative Care, Peter MacCallum Cancer Centre, Melbourne, VIC, Australia; 8 Department of Medical Oncology, Peter MacCallum Cancer Centre, Melbourne, VIC, Australia; 9 Sir Peter MacCallum Department of Medical Oncology, The University of Melbourne, Melbourne, VIC, Australia; 10 Department of Haematology, Royal North Shore Hospital, Sydney, NSW, Australia

**Keywords:** research ethics, oncology, cancer, equipoise, adaptive trial, platform trial, randomised controlled trial

## Abstract

Equipoise is usually considered a requirement for the ethical conduct of phase III randomised controlled trials. Notwithstanding theoretical critiques since the concept’s initial proposal, equipoise has remained an important moral consideration. In oncology, many research questions are now pursued via clinical trial designs that diverge in various ways from conventional, fixed-design, two-arm randomised controlled trials. In this article, we outline the challenges that these new designs pose for implementing equipoise. Specifically, we consider challenges posed by trials incorporating seamless designs, ‘immortal’ trials, and trials incorporating adaptive techniques. While we note that equipoise retains a central role in the ethical evaluation of conventional randomised controlled trials, we contend that its utility as a moral construct is challenged by more recent, ‘novel randomised controlled trial’ designs. Consequently, other ethical tools become important in considering the different types of risk that trial participants are exposed to in modern clinical trial designs.

## Introduction

It has always been the case that research begins with a question, or some degree of uncertainty about the way things are. During the 20th century, with the advent of randomised trials,^1^ the scientific and ethical importance of randomisation became more widely recognised, leading to discussion and debate regarding its purpose and meaning,^2^ and the idea of ‘equipoise’ began to be more formally regarded as an important ‘condition’,^3^ or at least ‘guiding principle’^4^ for the ethical conduct of phase III randomised controlled trials (RCTs).^3,5^ Initially, equipoise was theoretically conceptualised as uncertainty in the minds of individual physicians.^3^ In 1987, ethicist Benjamin Freedman introduced the notion of ‘clinical equipoise’, which he defined as a state of ‘genuine uncertainty’ about the relative potential benefits and risks of an experimental intervention, in comparison to the standard-of-care treatment, among the relevant ‘community of experts’.^6^ This collective understanding has subsequently come to dominate ethical discussion.

Equipoise has traditionally been considered an important component of the ethical justification for RCTs. This is on the basis that (1) ‘current’ patients are not knowingly disadvantaged by randomisation to an inferior treatment and (2) that trials are designed with the potential to maximally contribute to resolution of the clinical uncertainty under investigation^*^ (of benefit also to ‘future’ patients).^5,6^ However, in recent years, the cancer clinical trials landscape has evolved substantially, raising questions about the ongoing meaning and relevance of equipoise. Most of this debate has focused on the question of ‘whose uncertainty matters?’ when establishing if equipoise has been satisfied at the trial’s outset.^7^ For example, several recent phase III RCTs evaluating targeted therapeutics in non-small cell lung cancer (CodeBreak-200^8^ and LIBRETTO-431^9^) have been subject to public debate regarding equipoise^10,11^ and highlight tensions that may arise between (and among) different stakeholder groups (e.g. researchers, consumers, regulators).^12^

Even more fundamental challenges arise when attempting to operationalise equipoise in relation to trial designs which incorporate randomisation but diverge from conventional two-arm RCTs. Such ‘novel’ randomised trials include those that use ‘master’ protocols, ‘adaptive’ designs and/or other novel randomisation techniques.^13^ Consequently, it is not straightforward as to whether (or how) equipoise can or should apply to such trial designs. In this article, we consider how recent evolutions in drug development pipelines challenge traditional assumptions about the appropriate context for equipoise, highlight problems in applying equipoise to trials incorporating a range of novel methodological features, and consider implications for contemporary research ethics using the adult cancer trials landscape as an example.

## Equipoise in the context of modern clinical trials in oncology

The notion of equipoise arose as a result of the so-called ‘physician’s dilemma’,^6^ which became evident in the mid-20th century following the promotion of RCTs in clinical research, and described the challenge of conducting clinical trials while simultaneously prioritising individual patients’ best interests.^2,14^ It became clear around this time that it would only be palatable for physicians (and patients) to entertain trial participation when genuine uncertainty existed as to the relative merits of proposed study arms.^2^ Consequently, equipoise came to be seen as a theoretical ‘bridge’^15^ between routine care and clinical trials, allowing physicians to be comfortable about randomising patients to receive one or another therapy in the conduct of an RCT.

Although broadly accepted by both clinicians and researchers, the presumed ethical requirement for equipoise has been extensively critiqued, most prominently by ethicists Franklin Miller and Howard Brody, who have argued that it represents an inappropriate conflation of the relationship between investigators and trial participants as equivalent to that of physicians and patients during routine care.^15^ For Miller and Brody, investigators simply do not have the same ‘therapeutic obligation’ towards participants in clinical trials as clinicians do towards patients in routine care settings.^15^ This argument, that research and routine care are morally and professionally distinct,^15^ has been challenged on both ethical and practical grounds, in response to methodological, organisational and ‘cultural’ changes in healthcare. While these changes potentially apply to all areas of healthcare, they have been particularly salient in oncology. For example, leading cancer policy groups have explicitly advocated for the integration of clinical trials into treatment decision-making algorithms (e.g. the American Society of Clinical Oncology (ASCO)^16^ and the US-based National Cancer Comprehensive Network (NCCN), albeit such recommendations have also been subject to recent debate).^17,18^

While the integration of research and clinical care, if anything, provides support for the notion of equipoise, other recent changes in research and medicine also challenge its salience and utility. One such change is the progressive dissolution of traditional boundaries between different stages of drug development.^19^ Historically, clinical trials have been subdivided according to the degree of uncertainty regarding the investigational agent/s and the study’s primary objective/s (i.e. phase I – safety/dosing, II – efficacy, III – comparative efficacy/safety vs standard-of-care). Different types of clinical trials may therefore be viewed as unique epistemic tools for resolving varying amounts of uncertainty.^7,20^ According to this framing, philosopher Benjamin Djulbegovic describes equipoise as a particular type of uncertainty that occurs when ‘alternatives to be tested are clearly defined, and there is belief about “equal” prospect of superiority of one intervention over another which warrants RCTs to settle a dispute’.^20^ Over time, however, boundaries between different phases of clinical trials have become less distinct, complicating application of the concept.^19^ Consequently, whereas traditionally equipoise has been the expectation for ethical conduct of phase III RCTs but not first-in-human phase I studies (which we, and others,^20,21^ agree remains appropriate), it is increasingly difficult to determine whether and how to apply the concept to the increasingly liminal ‘in-between’ stages of drug development.^22^

Such liminality has become particularly notable in contemporary early trials of efficacy, the traditional phase II setting. Historically, high rates of attrition along drug development pipelines, and the relatively low rate of conversion from a ‘promising signal’ in a phase II trial to ‘success’ in the phase III setting, have both been recognised as problematic.^23^ In part, this is because the conduct of phase III RCTs is time-consuming as well as costly and burdensome to patients.^22^ These issues have become more and more salient with the veritable ‘explosion’ in volume of novel drug candidates (and combinations) in need of evaluation.^24^ Consequently, in recent years, various strategies have been proposed to improve the ‘positive predictive value’^22^ of phase II trials to forecast ‘success’ in the phase III setting. However, efforts to make phase II trials ‘more predictive’^22^ simultaneously raise a potentially troubling paradox for equipoise. As ethicists Spencer Hey and Jonathan Kimmelman have pointed out, if such efforts are ‘too successful’, such that the intervention is then perceived (rightly or wrongly)^25^ as more or less a ‘sure bet’, this may jeopardise the likelihood that equipoise can genuinely exist among the expert community at the outset of follow-on phase III RCTs.^22^ Moreover, if results of the phase II trial become very widely known, this may also threaten views about equipoise in the minds of referring clinicians and/or prospective trial participants (so-called ‘community equipoise’).^7^ While, according to Freedman’s conceptualisation of ‘clinical equipoise’,^6^ the morally relevant locus of uncertainty should be with the pertinent ‘community of experts’, uncertainty regarding equipoise among other stakeholders may also impact later-phase trials. As we have outlined previously, this may result in challenges to recruitment or lead to early asymmetric dropout among individuals randomised to standard-of-care comparators that they (or their referring physicians) suspect to be inferior.^12^

## Challenges posed by methodological innovations in trial design

In addition to shifts in the broad organisation of drug development, several more recent methodological approaches, which result in trials diverging in various ways from conventional RCT designs, pose new challenges for equipoise.

### The ‘blurred trial phase’ challenge

Seamless trial designs combine traditionally distinct trial phases within the one overarching trial protocol. For example, rather than conduct a phase II trial prior to instigation of a separate phase III trial, a single, composite trial approach is taken (i.e. a ‘phase II/III’ trial). Seamless designs encompass varying approaches to the use of participant data. In ‘operationally seamless but inferentially distinct’ trials, the one protocol is used but recruitment to and participant data from the earlier- versus later-phase components of the trial are analysed separately.^26^ In contrast, in ‘inferentially seamless’ trials, data pertaining to all or some participants enrolled in the earlier-phase component may also be carried over and contribute to the overall analysis.^26^

As such, while seamless designs may promote time and logistical efficiencies in drug development,^27^ they complicate the task of identifying when equipoise should apply, particularly with regard to inferentially seamless designs. As noted, equipoise may be viewed as a particular type of uncertainty, conventionally located at the outset of a phase III RCT, in which ‘it is estimated that there is about equal probability that one treatment is better than the other’.^20^ Prior to phase III and so earlier on in development, comparatively less is known about an investigational agent, and so exposure to ‘risks and potential benefits’^28^ in earlier-phase studies holds different moral weight than for phase III studies.^22^ As such, while uncertainty exists at the outset of a phase II trial, it would not usually be the kind of uncertainty that would be described as being ‘in equipoise’ about the relative merits (and/or disadvantages) of a few discrete alternatives.^20^ For seamless trials, particularly phase II/III trials which straddle this transition zone, it becomes difficult to appropriately locate (and be assured of) equipoise and communicate the implications of such uncertainties to prospective participants.

### ‘Immortal trial’ challenges

Trials incorporating adaptive platform or multi-arm multi-stage (MAMS) designs diverge from standard ‘A-vs-B’ RCTs in various ways. One key difference is the opportunity to incorporate new study arms throughout the trial, in a virtually ‘perpetual’^29^ manner, earning such trials the label ‘immortal study designs’.^30^ While flexibility to accommodate new research questions may be advantageous, trials that run over extended durations confront other challenges. This includes the importance of accounting for ‘population drift’,^29,31^ a phenomenon that can occur if the background standard-of-care treatment evolves throughout a trial’s lifespan such that the trial’s control arm becomes outdated.^31^ Population drift therefore introduces complexities for the ongoing maintenance of appropriate standard-of-care comparator/s,^32^ and challenges identification of the appropriate reference point for determining equipoise. That is, in a given trial, is equipoise (re)confirmed with each new study arm added, and if so, by whom (e.g. trialists, Institutional Review Boards (IRBs), regulators)? Such considerations may not be insurmountable, although they add considerably to the demands^33^ on those evaluating such trials, particularly if conducted over very long periods. Moreover, how different IRBs handle such proposals may well vary, with implications for assuring that necessary expertise is available to consider questions related to equipoise – for example, whether proposed additional arms are reviewed as ‘protocol amendments’ and go through an expedited, abbreviated committee review, or are subject to a full IRB review.

For adaptive platform and MAMS trials inclusive of common control arms, a further challenge relates to determining which portion of the control to use when conducting pairwise analyses, given that experimental arms may commence at staggered time-points throughout such trials.^34^ Use of ‘concurrent’ versus ‘non-concurrent’ control arm data remains subject to ongoing debate among biostatisticians (noting different implications for each, such as accounting for temporal confounders).^35^ For research ethics, this complicates attempts to locate the appropriate reference point for equipoise – that is, which component of the control arm is (or should) be considered as the comparator in attempts to evaluate the status of equipoise as each new arm is added?

### The ‘accumulating data’ challenge

A separate challenge that arises in trials incorporating adaptive methods is the so-called ‘accumulating data’^36^ challenge, which some would suggest poses a problem for equipoise as a consequence of the opportunity to modify the design of ongoing trials in response to evolving data trends. The question here is whether equipoise is maintained, or disrupted, if data have evolved to the extent that criteria for enactment of pre-specified methodological adaptation/s are met.^†^ The place of equipoise in adaptive trials generally has become one of the most hotly debated topics among researchers, biostatisticians, and ethicists, particularly in trials incorporating outcome-adaptive randomisation techniques.^36–44^ It may be argued that such debates arise from differing conceptualisations of how and when data becomes evidence,^36,45^ and when evidence is sufficient to disrupt equipoise. While some suggest that data reviewed during interim analyses in adaptive trials may not meet the epistemic threshold for equipoise disruption^46^ and so study continuation remains ethically justified, those with differing epistemological outlooks disagree.^47^

In considering equipoise and adaptive trials, philosopher Nicolas Fillion highlights the importance of appreciating variability in understanding and use of the word ‘evidence’.^36,48^ In particular, Fillion focuses on how this varies with different statistical inferential frameworks. Whereas in frequentist trial designs, at any given point a ‘dichotomy’ exists such that there is either sufficient evidence to reject or accept a hypothesis or not, this differs in trials designed with Bayesian statistical assumptions.^36^ In the latter, trends in accumulating data may be leveraged as evidence motivating refinement of an ongoing adaptive trial design; however, such ‘decisions [are made] without having reasons to conclude that a treatment is superior’ and therefore needing to stop the trial.^36^ Returning to the second so-called ‘epistemic’ feature of equipoise, this may be conceptualised as there remaining insufficient evidence to ‘convince’ the ‘expert community’.^‡^ Moreover, as ethicist Alex London argues using the example of outcome-adaptive randomisation, if the broader ‘community of experts’ beyond the trial remains uncertain, it may be argued that ‘clinical equipoise’ has not been disrupted.^46^

While adaptive designs have brought the so-called ‘accumulating data problem’^36^ to the fore, it is important to recognise that critics have long raised concerns about the status of equipoise following trial commencement (even in fixed-design trials). That is, how long can equipoise endure once data accrual begins in any type of clinical trial?^47^ To some extent the answer to this question is dependent on the type of uncertainty equipoise is understood to relate to. As philosopher Fred Gifford highlights, is the focus a mathematical question (e.g. ‘does treatment X cause outcome O with greater frequency (and extent) than does Y?’), or alternatively, a more ‘multi-dimensional’^49^ question (e.g. ‘is X a better treatment for these patients than is Y?’).^49^ We would suggest that equipoise is most usefully understood in the latter manner, that is, when mapped to the more clinically oriented uncertainty (which was arguably the original purpose of Freedman’s equipoise).^6^ If, however, we agree that equipoise is best applied to clinical questions and clinical uncertainty rather than purely ‘scientific’^49^ questions, additional challenges arise.^49^ First, clinical concerns require collection of more comprehensive metrics (e.g. safety, quality-of-life, patient-reported outcomes, and so forth). Second, it inevitably invokes value judgements about which outcome/s are important, which may vary among different stakeholders (e.g. clinicians, consumers, regulators). Being aware of these debates surrounding the relevant question that decisions regarding equipoise should focus on is helpful for appreciating why people may have different ideas about whether equipoise remains following trial adaptation/s.

## Equipoise and the ethical evaluation of ‘novel RCTs’

The notion of equipoise, and whether it should continue as a moral pre-requisite for RCTs, remains an ongoing topic of robust debate, particularly within the research ethics community.^6,15,47,50–54^ However, among clinical trialist communities, including in oncology, it must be acknowledged that equipoise remains an important ethical yardstick as well as the common language through which debates surrounding the justification of late-phase trials play out.^10,55,56^ To some extent, we support this desire to retain the concept – for example, we have argued elsewhere that good arguments exist for retaining the centrality of ‘clinical equipoise’^6^ in the ethical evaluation of conventional phase III RCTs.^12,57^ However, recent innovations in trial methodology (including those described and others, such as basket/umbrella^58^ and ‘*n*-of-1’^59^ trials) raise new questions about the possibility, construction, and practical application of equipoise in modern trials. While such trials may incorporate randomisation, they diverge from conventional two-arm, fixed-design RCTs and so complicate extrapolation of ‘clinical equipoise’^6^ as a moral construct. We contend that, while equipoise remains relevant to consider in relation to ‘novel RCT’ trials, it is likely that determining its status will be more complex in such settings. This is significant, as while satisfaction of equipoise alone is not a ‘sufficient’ condition for ethical trial design and conduct, it has nonetheless traditionally been the implicit (it is not, at times, explicit) default assumption among clinician and patient communities.

In this regard, it is noteworthy that several other ethical approaches have been proposed for the evaluation of clinical trials incorporating randomisation. While these approaches are sometimes presented as counter-arguments to the importance of equipoise, we suggest that they are most useful in circumstances in which the status of equipoise cannot be confidently established such as in the evaluation of ‘novel RCTs’. Here, we suggest a composite approach, which we refer to as a ‘toolkit’ of ethical concepts. We describe these briefly below. Table 1 provides more detail on each tool and its limitations (particularly if used in isolation). Table 2 offers a range of questions different stakeholders can consider when evaluating clinical trials.

**Table 1.** Suggested ‘toolkit’ for ethical evaluation of contemporary ‘novel RCT’ late-phase clinical trials in oncology.

‘Ethical toolkit’ components	Description of concept	Common critiques
‘Clinical equipoise’ As proposed by Benjamin Freedman (1987)^6^	Present when ‘maximum uncertainty’ exists between two discrete alternatives, with an RCT considered the best tool to resolve the uncertainty.^20^ Importantly, for ‘clinical equipoise’, Freedman locates the morally significant uncertainty amongst the relevant ‘community of experts’.^6 a^	• Conceptualised with reference to standard, two-arm, fixed-design RCTs^b^ (i.e. novel agent vs standard-of-care/control), hence difficulties applying to more recent types of trial which diverge from this design • Moral authority may be pragmatically challenged if stakeholders beyond the ‘community of experts’ perceive (rightly or wrongly) that equipoise is not present/has been lost (e.g. loss of ‘community equipoise’^7^ which may lead to difficulty in recruitment)^12^
‘Non-exploitation framework’ framework As proposed by Franklin Miller and Howard Brody (2002,^60^ 2003^15^)	Proposed as an alternative to equipoise, with two general criteria:^60^ (1) avoid exposure of participants to ‘excessive risks for the sake of scientific investigation’ (2) ensure participants comprehend they are ‘volunteering to participate in an experiment rather than receiving personalised medical care’ According to this approach, if a trial offers a reasonable potential risk/benefit balance and informed consent is present, then the trial is considered ethical.	• Determination of what constitutes ‘exploitation’ of participants in clinical research has long been subject to debate, as well as how to best balance risks to study participants vs potential benefits to society of undertaking clinical research • Criterion (2) may be undermined if participants conflate research and care (the therapeutic misconception), a well-described phenomenon in cancer cohorts^61^ • Determining what constitutes ‘excessive risk’ is intrinsically pluralistic, and likely to vary amongst and between different trial stakeholders (e.g. individuals confronting life-limiting illnesses with limited/no remaining therapeutic options may judge risk differently)^62^ • Some commentators have critiqued the potential for participants to be exposed to ‘less than standard’ care^63^
‘Ethical analysis of risk’ As proposed by Charles Weijer (2000)^28^	IRB is tasked with a ‘protective function’ (on the basis of beneficence and non-maleficence)^64^ of confirming ‘that study participation presents a favourable balance of potential benefits and risks …’. Advocates for a component analysis approach to risk evaluation in trial protocols, identifying aspects of trials which may be regarded as (1) ‘therapeutic procedures’ or (2) ‘non-therapeutic procedures’: 1) ‘therapeutic procedures’: must satisfy clinical equipoise. 2) ‘non-therapeutic procedures’: risk must be minimised and ‘be reasonable in relation to knowledge to be gained’.	• Still reliant on equipoise in part and involves extrapolation of equipoise beyond original conceptualisation (from evaluation of whole trials to components within trials) • As with the ‘non-exploitation’ framework, evaluation of risk is complicated by value pluralism as to what different stakeholders regard as ‘reasonable’ and/or ‘acceptable’ in regard to potential risks to study participants • Could be considered ‘demanding’ of IRBs (exhorts conducting literature review and consultation with other experts if necessary) and assumes expertise amongst the IRB to serve this function
‘Randomisation risks’ As proposed by Soren Holm, Jonathan Lewis, Rafael Dal-Ré (2023)^65^	Noting the overlapping nature of research and care, particularly in ‘fast moving’ research circumstances (the authors consider the SARS-CoV-2 pandemic as an exemplar), there is a need for ‘greater scrutiny’ regarding ‘randomisation risk’, that is, implications of randomisation to a study arm in which greater uncertainty exists regarding the status of equipoise. The authors advocate that particular consideration be given to ‘risk inherent in being allocated to a known or suspected inferior arm and ensuring’: (1) ‘this randomisation risk falls below the threshold for consentable risk’ (2) ‘that potential participants are fully informed about that risk’	• In the research ethics literature generally, whether or not there should be limits as to the magnitude and/or nature of risks that prospective trial participants should (or even can) consent to when considering clinical trial participation remains subject to debate (e.g. some might argue that imposing limitations in this area infringes on individual autonomy, whereas others may be dubious whether genuine informed consent even remains possible above certain risk thresholds). Individuals with life-limiting illness, such as cancer, may judge acceptability of risk differently,^62^ and this possibility would need to be accounted for during ethical review It is unclear to what degree trial participants understand and appreciate the implications of randomisation for themselves in standard RCT designs, let alone randomisation in more complex trial designs

a This in contrast to ethicist Charles Fried’s earlier conceptualisation of equipoise (1974), which placed the locus of uncertainty in the mind of individual physicians.^3^ Fried’s equipoise has subsequently been critiqued as ‘fragile’ and vulnerable to idiosyncrasy,^12^ with Freedman’s equipoise (1987) establishing itself as the dominant conceptual understanding.^6^

b While some have sought to apply equipoise to other trial phases, as noted, we find this less compelling given greater (and different) uncertainty applies earlier on in an agent’s developmental trajectory. Moreover, even when earlier-phase studies include multiple study arms, the purpose of doing so is not necessarily to facilitate the conduct of formal randomised comparisons as occurs in the phase III setting. Consequently, participants entering such trials would be consented as for the purposes of a phase I or II trial respectively including the willingness to submit to greater uncertainty regarding potential risks and/or benefits – if any – rather than with the expectation of equipoise between both study arms as for the outset of a phase III RCT. In addition, a separate challenge for single-arm trials relates to determination of the relevant (‘control’) comparator. This becomes difficult given the complexity of the contemporary cancer care landscape insofar as ‘treatment’ options (e.g. agents publicly reimbursed in one regulatory jurisdiction and not others, agents only available via self-funding, industry-funded ‘compassionate’ access schemes which open/close over time). For these reasons, it is our contention that equipoise operates best as an evaluative tool when applied to the phase III setting.

**Table 2.** Possible questions for different stakeholder groups to consider in applying ethical toolkit.

Stakeholder group	Possible questions
Clinicians	• Is the design of this trial more readily appropriate for evaluating against the equipoise requirement? (i.e. phase III RCT of a novel agent vs standard-of-care) • Am I satisfied the patient has appreciated the difference between participating in a clinical trial versus receiving routine care? ○ Has the patient appreciated the potential risks and/or known/expected burdens of participation? ▪ Related to the investigational agent? ▪ Related to logistical requirements of the study? • What are the possible ‘randomisation-related’^65^ risks associated with trial participation for this patient? ○ Are there any conceivable ‘opportunity costs’ to this patient should they choose to participate in this trial? ▪ In the event this patient is randomised to the standard-of-care/control arm, is the agent offered similar/equivalent from the local standard-of-care in the setting in which the patient is receiving treatment^a^? (particularly important to consider if the trial is being conducted internationally) ▪ If the standard-of-care/control arm is different to the local standard-of-care agent, has this been divulged to the patient considering trial participation (either in the PICF or verbally during the informed consent process)? ▪ Will participation in this trial have implications for eligibility for another trial, or for later-line standard-of-care^b^? ○ Have the ‘experimental’ agent/s under evaluation in this clinical trial received preliminary regulatory approval? ▪ If yes, and the agent is available locally, has the opportunity to access the agent off-trial been divulged to the patient? ▪ If yes, but the agent is not available locally, or is available locally at cost / via a ‘cost share’ or other ‘compassionate access’ scheme, has the potential opportunity to access the agent off-trial been divulged to the patient? • Are there any potential conflicts of interest relevant to disclose to prospective trial participants related to this trial? ○ For individual clinicians: Do I stand to gain professionally (e.g. academic credit) or in some other way by recruiting patients to this trial? ○ For clinical trial sites: Has the site committed to a recruitment target?
Prospective trial participants	• How does participating in this clinical trial differ from routine care? • What potential risks do I need to be aware of? ○ If I receive the experimental treatment/s? ○ If I receive the standard-of-care treatment/s? ○ Related to extra tests that I wouldn’t receive if having routine care? (e.g. potential complications of a tissue biopsy) • What known/expected additional burdens do I need to be aware of? ○ Related to the logistics of participating in this trial? (e.g. study schedule, additional tests) • Are there any foreseeable anticipated benefits that I would forego by choosing to participate in this trial? ○ If yes, am I still willing to participate in this trial? ○ What would be the alternative option/s to participating in this trial? • Will/have there be any changes to the design of the trial over time? ○ If yes, is it possible that some of the experimental treatment arms could be stopped during the trial? If I were allocated to that arm, what would happen to my involvement in the trial beyond that point?
Institutional review boards	• Is the design of this trial more readily appropriate for evaluating against the equipoise requirement? (i.e. phase III RCT of a novel agent vs standard-of-care) ○ If yes, is there an ‘honest, professional disagreement’ apparent amongst the relevant ‘community of experts’^6^ regarding the trial’s central question? (i.e. ‘clinical equipoise’ appears present) ○ If this is a multi-arm trial (e.g. platform trial), is there sufficient uncertainty (regarding potential harms/benefits) for each pairwise comparison (i.e. study arm vs control) that equipoise appears apparent amongst the ensemble of study arms?^66^ ▪ If this is an adaptive platform trial in which new study arms are being added (and/or the control arm is updated)^29,66^over time, has equipoise been re(checked) for each new pairwise comparison/s that emerge throughout the duration of the trial?^66^ • Are the interventional +/− control arm/s of this study scientifically justifiable?^28^ ○ Is the information provided in the trial protocol sufficient to establish this? (do we need to conduct a literature review and/or consult external expert opinion?)^28^ • Is this likely to be a trial running over an extended duration? ○ If yes, how will ongoing appropriateness of the control arm be assured and/or (re)checked?^32^ • Have any identified potential risks and/or known/expected burdens to participants been minimised as much as possible?^28^ ○ Is exposure to risk proportionate to the anticipated value of knowledge to be generated?^28^ ○ Has there been consumer input? • For adaptive trials, has the possibility of changes to trial design throughout the running of the trial been sufficiently explained in the consent documents? ○ Has there been consumer input? • Is the rationale for this trial underpinned by a biomarker-dependent hypothesis? ○ If yes, is the locus of scientific uncertainty in this trial about a novel biomarker-directed therapeutic, or about a novel diagnostic technology (for identification of the relevant biomarker-specific population), or both?^66^ If the latter, are both types of uncertainty outlined in the consent documents?

a For clinical trials running over very extended durations, given the potential for population drift regarding background standard-of-care, determining whether the standard-of-care/control arm is *still* relevant to the local setting has significance as well.

b This may be particularly significant for individuals with rapidly progressive and/or poor prognosis tumour types in which the likelihood of being well enough to receive multiple lines of therapy is low.

The ‘non-exploitation’ framework, advanced by Miller and Brody (2003),^15^ consists of two main criteria: (1) that exposure of participants to ‘excessive risks’ for the sake of research should be avoided and (2) that participants should understand they are ‘volunteering … [for] an experiment rather than receiving personalised care’.^15,60^ Consequently, providing a trial offers a reasonable potential risk–benefit balance and informed consent is present, the trial may be considered ‘ethical’ per this framework. An alternative proposal, advanced by ethicist Charles Weijer, is the ‘ethical analysis of risk’ approach (2000).^28^ For Weijer,^28^ tasking IRBs with a ‘protective’ role is key, as well as the importance of ensuring that ‘study participation presents a favourable balance of potential benefits and risks’. This proposal also involves a ‘component analysis’ approach to evaluation of potential risk/s, which entails identifying elements in the trial protocol which may be viewed as ‘therapeutic’ versus ‘non-therapeutic’.^28^ Whereas Weijer argues that for ‘therapeutic procedures’ clinical equipoise must still be satisfied, he suggests that for ‘non-therapeutic procedures’, risk must be minimised and ‘reasonable in relation to knowledge to be gained’.^28^

A more recent idea focuses on the identification of possible ‘randomisation risks’ of trial participation.^65^ This approach has been proposed by ethicists Soren Holm et al., prompted by their consideration of two ‘novel RCTs’ conducted during the COVID-19 pandemic of an anti-viral agent.^65^ Specifically, their proposal is to distinguish ‘randomisation risks’ that occur as a result of participation in research but differ from other, ‘standard research-related’ risks.^65^ That is, ‘randomisation risks’ may be noted as ‘risk[s] inherent in being allocated to a known or suspected inferior arm’ of a clinical trial.^65^ In contrast, ‘standard research-related risks’ are (1) those that result from uncertainty surrounding benefit and/or harms of the experimental intervention (but not so much the relative benefits or harms of being assigned to one arm or another, as these by definition are not known) or (2) those arising from additional non-care related investigations.^65^ For ‘novel RCT’ designs, in which there may be greater uncertainty as to how to operationalise equipoise (and so be confident about its status at the outset of a trial), it becomes increasingly important to consider possible implications for participants randomised to each of the proposed study arms. In such circumstances Holm et al. advocate for ‘closer scrutiny’ of the acceptability of risks borne by study participants, as well as transparency in informed consent processes about such risks (Table 1). While in oncology, it is hard to imagine that patients (or their referring clinicians) would be willing to accept randomisation to a ‘known’ inferior study arm,^56,67^ the idea of ‘randomisation risk’ may also be salient where equipoise is simply more difficult to confirm due to a trial’s design characteristics.

A common thread among each of the approaches outlined above is the recognition that exposure to risk as a consequence of clinical trial participation, whether research- or randomisation-related, is not ethically problematic in-and-of-itself. Research involving human experimentation always requires some balancing of the interests and risks borne by current participants as well as those borne by future recipients of a clinical intervention, with obligations to both types of stakeholders. For future, recipient, populations,^§^ there is the obligation to conduct trials with the greatest epistemic capacity to resolve the underlying community uncertainty (of which, others have sought to demonstrate empirically^‖^ being in a state of equipoise facilitates).^68^ For research participants, risk communication is critically important because individuals’ assessments of the potential risk–benefit balance of trial participation will inevitably vary and need to be weighed against the risks and opportunities of choosing not to participate in a given trial. Importantly, for an individual to make an informed decision, they should be made aware of any possible ‘opportunity costs’ which may be associated with participation. While this will vary widely depending on an individual’s specific circumstances, it could mean, for example, that part of facilitating autonomy involves informing prospective trial participants about any avenues that may offer a means of accessing the novel agent off-trial (e.g. pharmaceutical company ‘drug familiarisation’ programmes, including those requiring ‘self-funding’).^69,70^

While there may be ‘scientific’ and other reasons to continue to generate trial-based evidence about a specific agent, even where equipoise cannot be confidently determined,^71^ such as when stipulated by regulatory agencies to extend follow-up or safety data, this may not be compelling for individual patients or clinicians to pursue trial enrolment. As such, when uncertainty regarding the status of equipoise is recognised, but a trial is nonetheless granted ethical approval, it behoves IRBs to explain the nature of this uncertainty about equipoise – in particular that equipoise in this context is contested and relatively unclear compared to standard RCTs. It is also important to explain any potential specific risks of being randomised to particular study arms.^57^ At least for some individuals, it may be that lack of assurance regarding equipoise is ‘material’^72^ information for a decision not to participate in a trial,^67^ while for others, the level of uncertainty about equipoise might be tolerable.

Such scenarios highlight the challenge that arises when clinical trials are perceived, on one hand, as treatment opportunities and a way of accessing promising novel therapeutics,^73^ while also serving the public health goals of contributing to knowledge for the benefit of future patient populations.^61,74^ Given the global stage upon which clinical trials are conducted in oncology, in which early data (and flow-on regulatory/reimbursement decisions) from one clinical trial may have implications for ongoing trials elsewhere,^65^ issues surrounding the satisfaction of equipoise, as perceived by various stakeholder groups, are likely to become increasingly challenging over time.

## Conclusion

While modern medicine has undoubtedly benefitted from innovation in the science of clinical trial design,^30^ each methodological development has introduced new moral trade-offs. In this context, it is important to remember that the acceptability of randomisation in trials generally may be viewed as only ever permissible via ‘fragile social consensus’,^22^ and the ability to conduct research is contingent upon preservation of trust among potential trial participants, and within the scientific (and broader) community. Consequently, there is a pressing need for stakeholders engaged in the design and conduct of RCTs with novel design features to reconsider their ethical obligations. First, it is important to be transparent when there is uncertainty about equipoise (this is relevant to both conventional and ‘novel RCTs’).^57^ Second, it is important to take into account a broader range of ethical considerations when evaluating ‘novel RCTs’, where equipoise is uncertain or clearly not present.^55^ In these circumstances, it becomes necessary to also employ other evaluative ‘tools’, to determine whether randomisation is ethically acceptable considering the specific implications it would have for participants in the proposed trial in question.

## Notes

* In some cases, (2) is noted as a second, ‘epistemic’, feature of equipoise, namely that ‘a successful trial should be convincing enough to resolve the dispute among clinicians’.^6^ We agree that RCTs should be designed for maximal epistemic potential in the spirit of scientific excellence and social value, as well as to responsibly steward limited economic and patient resources.^5^ To this end, this so-called ‘condition’ for clinical equipoise^21^ protects both ‘current’ and ‘future’ patients by discouraging the design of RCTs with inappropriate comparators.^32^ However, we would also note that evidentiary thresholds for what may be considered ‘convincing enough’^6^ vary widely (and so, attenuates any claim to this feature being interpreted as ‘normative’ guidance).^36^ In some cases, it may be true that the results of a single (well-designed, markedly ‘positive’ or ‘negative’) RCT comprehensively resolve a clinical controversy – so-called ‘game-changers’. However, in many (if not most) other cases, it is more likely that results of a single RCT will simply ‘advance the needle’ further, noting the potential statistical ‘fragilities’ of individual RCTs^75^ with further trials (and meta-analyses) required to fully convince the expert (and broader) community.^36^

† Importantly, this is quite a different scenario to a situation in which ‘stopping’ criteria are met during an interim analysis of a given (adaptive or fixed-design) randomised trial. In such scenarios, ongoing conduct of the trial is terminated on the basis that equipoise has been disrupted, and it is no longer ethically justified to continue the trial given pre-specified criteria for efficacy, futility or safety have been met.

‡ While, as noted, from a philosophy of science perspective what is ‘convincing enough’^6^ is intrinsically pluralistic, it should be noted that even from a statistical methodology perspective, there are also a range of potential pitfalls to over-reliance on results of interim analyses in different types of adaptive trials, described extensively elsewhere.^76^ As one example, Sydes et al. highlight the importance of interpreting interim analyses on the basis of what they are – interim data which ‘reinforces the need to continue randomisation to gain stronger evidence for the definitive primary outcome measure’ –when describing how ‘investigator assumptions’ were handled during conduct of STAMPEDE, a landmark adaptive platform trial in prostate cancer.^77^

§ Arguably, this obligation is also towards current populations, beyond those participating in the trial, on the basis of a commitment to the conduct of research which maximises social and scientific value and uses (often limited) resources productively.

‖ At least in the context of conventional RCTs.
